# Duration of DKA and Insulin Use in People with and Without SGLT2 Inhibitor Medications

**DOI:** 10.3390/medicines12030021

**Published:** 2025-08-19

**Authors:** Yeung-Ae Park, Anya Kitt Lee, Rahul D. Barmanray, Frank Gao, Spiros Fourlanos, Chris Gilfillan

**Affiliations:** 1 Department of Diabetes and Endocrinology, Royal Melbourne Hospital, Melbourne 3050, Australia; 2 Department of Medicine, Royal Melbourne Hospital, University of Melbourne, Melbourne 3050, Australia; 3 Department of Endocrinology and Diabetes, Eastern Health, Melbourne 3128, Australia; 4 Monash School of Medicine, Monash University, Melbourne 3168, Australia; 5 Department of Diabetes and Endocrinology, Western Health, Melbourne 3021, Australia; 6 Australian Centre for Accelerating Diabetes Innovations (ACADI), University of Melbourne, Melbourne 3010, Australia; 7 Department of Medicine, Austin Health, University of Melbourne, Melbourne 3084, Australia; 8 Department of Endocrinology, Austin Health, Melbourne 3084, Australia

**Keywords:** diabetes, diabetic ketoacidosis, insulin resistance, sodium–glucose co-transporter 2 inhibitors, treatment outcome

## Abstract

Background/Objectives: Sodium–glucose co-transporter 2 inhibitors (SGLT2i) are associated with increased rates of diabetic ketoacidosis (DKA). The difference in the management and outcomes of SGLT2i-associated DKA (SGLT2i DKA) from non-SGLT2i-associated DKA (non-SGLT2i DKA) remains unclear due to a lack of specific reporting on dextrose and insulin. This study aims to compare the management and outcome of SGLT2i and non-SGLT2i diabetic ketoacidosis. Methods: In this retrospective cohort study, patients admitted to the Intensive Care Unit (ICU) for diabetic ketosis between 1 January 2020 to 31 December 2021 at a tertiary hospital were identified. For each SGLT2i diabetic ketosis, two non-SGLT2i diabetic ketosis admissions closest to the SGLT2i admission date were evaluated for comparison. Clinical data including biochemistry, ICU length of stay (LOS), time to normalize acidemia and ketonemia, dextrose and insulin requirements, were evaluated. Results: In the SGLT2i group (*n* = 30), there were 22 DKA and 8 diabetic ketosis cases; in the non-SGLT2i group (*n* = 60), there were 54 DKA and 6 diabetic ketosis cases. SGLT2i DKA (*n* = 22) required 62% greater total insulin (154 [117–249] vs. 95 [59–150] units; *p* = 0.004), which remained statistically significant after weight adjustment (*p* = 0.02), and longer ICU LOS (52 [42–97] vs. 39 [23–68] hours; *p* = 0.01) compared to non-SGLT2i DKA (*n* = 54), despite a comparable time to DKA resolution (22 [15–35] vs. 20 [15–35] hours; *p* = 0.91). In the intercurrent illness subgroup analysis, neither total insulin dose nor ICU LOS remained statistically significantly different between SGLT2i (*n* = 16) and non-SGLT2i DKA (*n* = 21). The majority of cases received 10% dextrose and variable rate intravenous insulin infusion (VRIII). Conclusions: The greater insulin requirement in SGLT2i DKA compared to non-SGLT2i DKA may be explained by the greater proportion of precipitating intercurrent illnesses and demographic differences in SGLT2i DKA, highlighting that SGLT2i DKA (predominantly comprising T2D) and non-SGLT2i DKA (predominantly comprising T1D) represent distinct clinical entities. Our findings in comparison to the literature imply that in SGLT2i DKA, the need for prolonged IV insulin infusion may be reduced through intensive management using intravenous 10% dextrose and VRIII. Prospective studies are warranted to evaluate the efficacy of different management strategies for SGLT2i DKA.

## 1. Introduction

Sodium–glucose co-transporter 2 inhibitors (SGLT2i) have emerged as highly effective therapies for type 2 diabetes (T2D) given their glycemic efficacy, effects on weight loss and well-recognized cardio- and reno-protective benefits [1]. Key consensus international guidelines recommend SGLT2i use in people with T2D who have established cardiovascular or kidney disease or multiple risk factors for cardiovascular disease [2,3].

In 2015, the United States Food and Drug Administration warned that SGLT2i use is associated with diabetic ketoacidosis (DKA) [4]. The use of SGLT2i in patients with T2D has been associated with an increased risk of developing DKA by approximately two-fold compared to non-SGLT2i users [5,6], with an incidence rate of 1.02/1000 for SGLT2i users and 0.69/1000 for SGLT2i non-users [7]. SGLT2i initiation in hospital has similarly been associated with numerically higher ketoacidosis rates (0.210 per 100 person-years) compared with control (0.140 per 100 person-years) [8]. Traditionally, DKA presents with hyperglycemia and ketoacidosis; however, with SGLT2i use, patients may present with euglycemic DKA [9].

Although the increased incidence, demographic and initial biochemical differences between SGLT2i DKA and non-SGLT2i DKA have been described, there is a paucity of studies evaluating the management practices and comparison of outcomes between SGLT2i DKA and non-SGLT2i DKA. The duration of SGLT2i DKA has been previously described to be significantly greater compared to non-SGLT2i DKA [10,11] or all-cause DKA [12]. Similarly, SGLT2i DKA has been associated with longer intensive care unit (ICU) length of stay (LOS) compared with non-SGLT2i DKA [10,13,14]. However, a lack of reported data on intravenous (IV) dextrose concentrations and insulin regimens has limited the assessment of optimal fluid and insulin management practices for SGLT2i DKA.

We aimed to compare the management and outcomes of SGLT2i DKA and ketosis with non-SGLT2i DKA and ketosis, including the amount of insulin required, IV fluid management with dextrose specifications, time to DKA resolution, and ICU LOS.

## 2. Material and Methods

This retrospective cohort study of patients admitted to the ICU for diabetic ketosis over two years between 1 January 2020 and 31 December 2021 at a tertiary hospital network was approved by the Office of Research and Ethics at Eastern Health. All patients admitted to the ICU with SGLT2i diabetic ketosis were included in the study. The patients were identified through International Classification of Diseases codes for DKA. Individuals with SGLT2i DKA (beta-hydroxybutyrate [BOHB] ketones > 0.6 mmol/L; pH < 7.30) or SGLT2i diabetic ketosis (BOHB ketones > 0.6 mmol/L; pH ≥ 7.30) were included in the SGLT2i group. Patients without confirmed ketonemia were excluded. SGLT2i usage was determined based on documentation as a current medication in the admission medical records and through pharmacy medication reconciliation. For each case of SGLT2i DKA or SGLT2i diabetic ketosis, two non-SGLT2i DKA or diabetic ketosis admissions that occurred closest to the index SGLT2i-associated admission date were evaluated for comparison. Where cases of SGLT2i DKA or SGLT2i diabetic ketosis occurred on consecutive or identical admission dates, the next closest non-SGLT2i DKA or diabetic ketosis admissions to the index SGLT2i admission dates were compared. There were no exclusion criteria. Clinical and laboratory information, including patient demographics and diabetes-related clinical history, were collected from the medical record. Precipitating intercurrent illnesses were defined as infection, acute myocardial infarction, pancreatitis, surgery, and steroid use.

The main outcome measures were total insulin requirement during diabetic ketosis or DKA treatment, time taken to normalize acidemia (pH 7.35–7.45) and ketonemia (BOHB ketone < 0.3 mmol/L), and ICU LOS. Other outcomes included IV fluids use, including type, infusion rates, and the total amount used. Recurrence of ketonemia was defined as BOHB ketones > 1.0 mmol/L after falling to <0.3 mmol/L.

An identical protocol was utilized for SGLT2i and non-SGLT2i DKA. Per protocol, if blood glucose level (BGL) was <15 mmol/L, 10% dextrose was commenced at 83 mL/h, and the insulin infusion rate was titrated according to the blood glucose level.

Statistical analysis was conducted using IBM SPSS statistics for Windows, Version 24.0 (Armonk, NY, USA: IBM Corp.). Variables were checked for distribution and normality prior to analysis. Continuous variables are presented as mean  ±  standard deviation or medians (interquartile range [IQR]). Fisher’s exact tests and binary logistic regression were utilized for categorical variables. Parametric and non-parametric tests were utilized to assess the associations between continuous variables. All statistical tests were 2-tailed, and statistical significance was set at *p* < 0.05.

## 3. Results

### 3.1. Baseline Demographics of the Study Population

Twenty-two cases of SGLT2i DKA and eight cases of SGLT2i diabetic ketosis were identified in the SGLT2i group (*n* = 30). For comparison, 54 cases of non-SGLT2i DKA and six cases of non-SGLT2i diabetic ketosis were analyzed in the non-SGLT2i group (*n* = 60). The SGLT2i group consisted of older people (53.0 [47.0–62.3] vs. 31.0 [24.3–48.0] years; *p* < 0.001) with higher body mass indexes (BMI) (31.1 [26.5–35.6] vs. 24.2 [21.7–27.2] kg/m^2^; *p* < 0.001) than the non-SGLT2i group. The SGLT2i group consisted largely of people with T2D (83%), whereas the non-SGLT2i group only consisted of people with type 1 diabetes (T1D) (100%). Three patients with SGLT2i (10%) had a diagnosis revised from T2D to latent autoimmune diabetes in adults (LADA) due to diabetes-related autoantibody positivity during admission.

### 3.2. Initial Biochemistry and Presentations

The SGLT2i group had significantly lower glucose (14.1 [9.9–26.5] vs. 27.1 [23.9–36.6] mmol/L; *p* < 0.001) and ketone levels (4.1 [3.6–5.5] vs. 6.2 [4.5–7.0] mmol/L; *p* < 0.001), and higher serum bicarbonate levels (14.0 [11.0–19.0] vs. 9.5 [6.0–13.0] mmol/L; *p* = 0.003) on initial presentation compared to the non-SGLT2i group (Table 1). Precipitating intercurrent illnesses were more prevalent in the SGLT2i group than in the non-SGLT2i group (73% vs. 42%; *p* = 0.007). The most common precipitant for the SGLT2i group was infection (60%), whilst insulin omission (53%) was the most common precipitant of the non-SGLT2i group, followed by infection (37%).

### 3.3. Management and Outcomes of DKA

**Figure 1:**
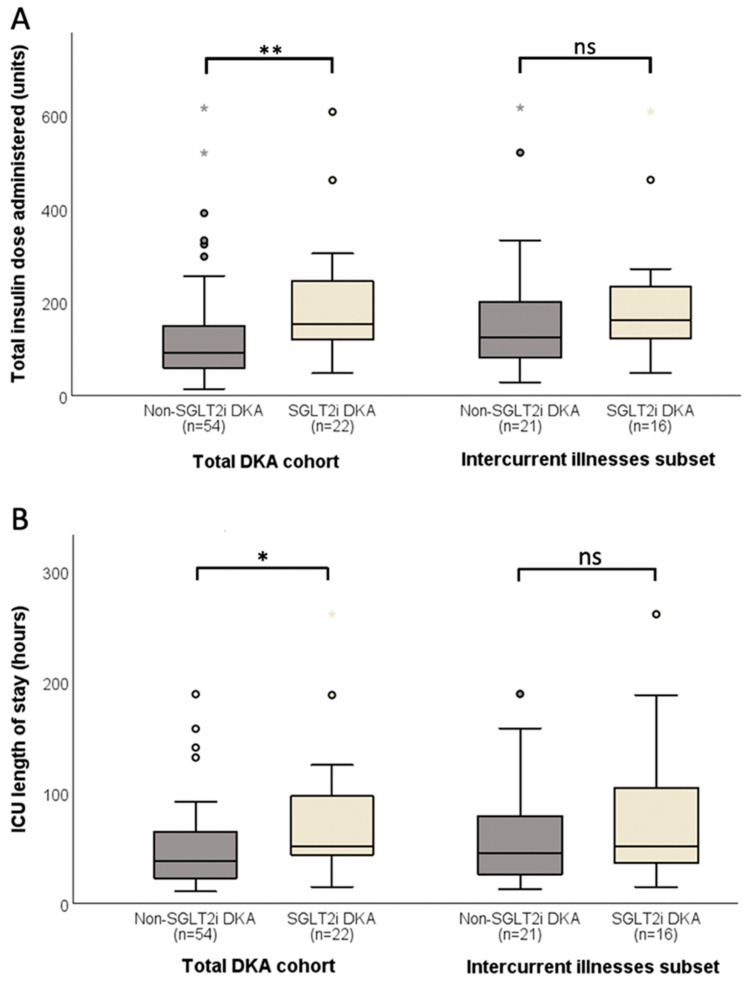
Comparison of (**A**) total insulin dose required for DKA resolution and (**B**) ICU LOS between SGLT2i DKA and non-SGLT2i DKA in the total DKA cohort and the subgroup with intercurrent illnesses. *, *p* < 0.05; **, *p* < 0.01; ns, not significant; DKA, diabetic ketoacidosis; ICU, intensive care unit; LOS, length of stay; SGLT2i, sodium–glucose co-transporter 2 inhibitors.

The SGLT2i group (*n* = 30) required significantly greater IV insulin (150 [107–228] vs. 89 [55–147] units; *p* = 0.004) and had longer ICU LOS (56 [43–101] vs. 40 [23–67] hours; *p* = 0.002) than the non-SGLT2i group (*n* = 60) despite comparable time to ketonemia and acidemia resolution (Table 2). SGLT2i DKA (*n* = 22) required 62% greater total insulin (154 [117–249] vs. 95 [59–150] units; *p* = 0.004), which remained statistically significant after adjustment for weight (1.87 [1.45–2.38] vs. 1.30 [0.75–1.96] units/kg; *p* = 0.02), and longer ICU LOS (52 [42–97] vs. 39 [23–68] hours; *p* = 0.01) compared to non-SGLT2i DKA (*n* = 54) (Figure 1), despite a comparable time to DKA resolution (22 [15–35] vs. 20 [15–35] hours; *p* = 0.91). In one case of SGLT2i DKA and four cases of SGLT2i diabetic ketosis, subcutaneous insulin was utilized without IV insulin. There was no statistically significant difference in the initial BOHB ketone levels between the individuals managed with IV vs. subcutaneous insulin (5.9 [4.0–6.8] vs. 3.9 [3.5–4.0] mmol/L; *p* = 0.08). Compared to the subcutaneous insulin subset of SGLT2i group (*n* = 5), IV insulin subset of the SGLT2i group (*n* = 25) required a greater amount of insulin (151 [107–253] vs. 36 [31–61] units; *p* = 0.001), which remained statistically significant after adjustment for weight (1.80 [1.11–2.38] vs. 0.58 [0.34–0.80] units/kg; *p* = 0.01), to achieve comparable resolution of ketonemia (19 [15–33] vs. 32 [14–46] hours; *p* = 0.45). Variable rate intravenous insulin infusion (VRIII) was utilized in all patients managed with IV insulin as per protocol.

There was no significant difference in the time taken for normalization of pH (11 [6–16] vs. 10 [6–14] hours; *p* = 0.94) and BOHB ketones (22 [15–35] vs. 20 [15–32] hours; *p* = 0.91) between SGLT2i (*n* = 22) and non-SGLT2i DKA (*n* = 54). In the subgroup analysis of precipitating intercurrent illnesses in SGLT2i (*n* = 16) and non-SGLT2i DKA (*n* = 21), neither total insulin dose (162 [117–247] vs. 125 [71–249] units; *p* = 0.17) nor ICU LOS (52 [36–108] vs. 45 [26–86] hours; *p* = 0.33) remained statistically significant (Figure 1). In the subset analysis by four groups of non-SGLT2i DKA without intercurrent illness (*n* = 33), non-SGLT2i DKA with intercurrent illness (*n* = 21), SGLT2i DKA without intercurrent illness (*n* = 6) and SGLT2i DKA with intercurrent illness (*n* = 16), the total insulin dose and ICU LOS were significantly different between the groups (70 [56–137] vs. 125 [71–249] vs. 141 [112–229] vs. 157 [133–231] units; *p* = 0.01) and (34 [21–56] vs. 45 [26–86] vs. 54 [48–74] vs. 52 [36–108] hours; *p* = 0.02), respectively. In those without intercurrent illnesses, the total insulin dose (*p* = 0.03) and ICU LOS (*p* = 0.02) were significantly greater in SGLT2i DKA compared to non-SGLT2i DKA. In those with intercurrent illnesses, the total insulin dose (*p* = 0.18) and ICU LOS (*p* = 0.34) were no longer statistically significantly different between SGLT2i DKA and non-SGLT2i DKA. In non-SGLT2i DKA, there was a trend toward greater total insulin dose (*p* = 0.09) and ICU LOS (*p* = 0.07) in those with intercurrent illnesses compared to those without. In SGLT2i DKA, the total insulin dose (*p* = 0.69) and ICU LOS (*p* = 1.00) were similar between those with intercurrent illnesses and those without.

The most common dextrose concentration used was 10% dextrose, used in 83% in the SGLT2i group and 93% in the non-SGLT2i group. The two groups had no difference in the total dextrose amount or total IV fluid volume administered (Table 2). Higher mortality was observed with the SGLT2i group compared to the non-SGLT2i group (*n* = 3 [10%] vs. *n* = 0 [0%]; *p* = 0.04) in the setting of concurrent illnesses (bowel ischemia, metastatic breast cancer and stroke).

### 3.4. Complications of DKA Management

Hypoglycemia occurred less frequently in the SGLT2i group compared to the non-SGLT2i group but was not significantly different (13% vs. 23%; *p* = 0.521). There were no vascular complications, such as thrombophlebitis, even in those managed with 10% dextrose. Hypokalemia was common in both groups, but no arrhythmias related to hypokalemia occurred.

### 3.5. Management Post DKA Resolution

Recurrent ketonemia occurred in over one-quarter of cases and was similar in both groups (Table 2). The recurrence of ketonemia was not correlated with ICU LOS (*p* = 0.58), diabetes duration (*p* = 0.34), initial biochemistry (glucose [*p* = 0.59], ketones [*p* = 0.97], pH [*p* = 0.74], bicarbonate [*p* = 0.17]), time to ketonemia resolution (*p* = 0.22), time to acidemia resolution (*p* = 0.27), total insulin dose (*p* = 0.95), c-peptide (*p* = 0.94), hemoglobin A1C (*p* = 0.57), or premorbid total daily insulin dose (*p* = 0.85). Greater total dextrose (233 [151–329] vs. 128 [75–221] g; *p* = 0.008) and IV fluid volume (7.5 [5.0–9.5] vs. 5.3 [3.0–6.7] L; *p* = 0.002) were observed in individuals with recurrent ketonemia compared to those without, reflective of the IV dextrose treatment following the recurrence of ketonemia. In the SGLT2i group, 21 (70%) were discharged with subcutaneous insulin, with 12 of them being new to insulin therapy. SGLT2i was ceased in all SGLT2i DKA and SGLT2i diabetic ketosis; however, consideration of recommencement was advised in those with identified and preventable precipitants and the ability to comprehend and follow SGLT2i action plans.

## 4. Discussion

Our results demonstrate for the first time that the greater prevalence of intercurrent illnesses is a potential explanation for the higher insulin requirement and longer ICU LOS observed in SGLT2i DKA compared to non-SGLT2i DKA. Inflammation is an important factor in the pathogenesis of insulin resistance in T2D [15], via pro-inflammatory cytokines in adipose tissue, skeletal muscle and liver [16]. Similarly, during infection and sepsis, insulin resistance is promoted in hepatocytes via interleukin (IL)-1beta, tumor necrosis factor and IL-6 and gluconeogenesis in the liver [17] via a systemic neuroendocrine response, including the release of cortisol, norepinephrine and epinephrine [18]. These processes theoretically affect all individuals with diabetes regardless of their underlying diabetes type. In other situations of physiological stress, such as myocardial infarction, acute insulin resistance has been demonstrated even in patients without diabetes [19]. The loss of statistical significance in total insulin dose between the SGLT2i and non-SGLT2i DKA cohorts with intercurrent illnesses, compared to the overall respective cohorts, was primarily driven by the increase in insulin requirements in the non-SGLT2i group with intercurrent illnesses. Our results suggest that even in younger and lean individuals with T1D, the effect of intercurrent illnesses such as infection and inflammation in insulin resistance is profound, thus requiring similar insulin requirement and ICU LOS as those with SGLT2i DKA, which typically represent the comorbid individuals with T2D and baseline insulin resistance precipitated by intercurrent illnesses. In contrast, the SGLT2i group showed only a minimal increase in insulin requirement in the setting of intercurrent illness, likely due to their baseline insulin-resistant state.

The lower initial glycaemia of the SGLT2i group compared to the non-SGLT2i group is well known and attributed to the glycorusic effects of SGLT2i [20]. The higher initial serum bicarbonate level of the SGLT2i group compared to the non-SGLT2i group in our cohort has been similarly observed by Umapathysivam et al., although statistical significance was not met [11]. Despite presenting with less severe initial hyperglycemia, ketonemia and acidemia, the SGLT2i group required a significantly greater amount of insulin to achieve a comparable resolution time of acidemia and ketonemia compared to the non-SGLT2i group. The explanation for this observation is likely multifactorial: (i) a higher proportion of intercurrent illness in SGLT2i DKA, (ii) demographic differences with the SGLT2i group consisting of largely patients with T2D with baseline insulin resistance than the non-SGLT2i group, (iii) delayed drug clearance with the half-life of SGLT2i being approximately 12 h, and (iv) pancreatic alpha cell secretion of glucagon and consequent hyperglucagonemia from SGLT2i [21], requiring treatment with greater insulin for ketone clearance compared to non-SGLT2i DKA. The differences observed between these cohorts reflect underlying demographic variations, highlighting that patients typically affected by SGLT2i DKA (primarily those with T2D) and non-SGLT2i DKA (primarily those with T1D) represent fundamentally different populations, rather than being solely attributable to the use of SGLT2i medication. The SGLT2i cohort included three patients whose diagnoses were revised from T2D to LADA, demonstrating that SGLT2i DKA may serve as an opportunity to reassess and refine the underlying diabetes diagnosis.

Greater ICU LOS was observed in SGLT2i DKA compared to non-SGLT2i DKA, with the difference disappearing when only comparing those with intercurrent illnesses. Similarly to our findings on insulin requirement, whilst ICU LOS numerically increased in non-SGLT2i DKA with intercurrent illnesses compared to those without, ICU LOS remained similar in SGLT2i DKA with intercurrent illnesses compared to those without. This suggests that the significant difference observed in ICU LOS between the overall cohorts of SGLT2i and non-SGLT2i DKA is primarily due to the differential prevalence of intercurrent illnesses. Our findings of greater ICU LOS in SGLT2i DKA align with several studies [10,13,14]; however, conflicting data exist [7]. Hamblin et al. [7] did not find longer ICU LOS in SGLT2i DKA, however the comparator group was non-SGLT2i DKA in T2D, which better resembles the demographic characteristics of the SGLT2i DKA group than the non-SGLT2i DKA in T1D, and the ICU LOS was calculated in days rather than hours, reducing the ability to detect small differences. Overall, the greater LOS and mortality in SGLT2i DKA in our study may reflect the underlying severity of intercurrent illnesses and the demographic differences between these cohorts rather than the DKA itself, given the comparable time to DKA resolution.

In contrast to previous studies describing the longer duration of SGLT2i DKA compared to non-SGLT2i DKA [10,11] or all-cause DKA [12], our study demonstrated no significant difference in time to ketonemia or acidemia resolution between the SGLT2i and non-SGLT2i groups. We postulate that this is due to the intensive insulin treatment enabled by a higher concentration of 10% dextrose. Improvement of intractable metabolic acidosis in SGLT2i DKA has been demonstrated after switching from 5% dextrose to high-calorie glucose infusion, such as 10% dextrose in case reports [22,23]. In a proposed SGLT2i DKA treatment algorithm [20], Chow et al. stated that a 10% dextrose infusion is preferable and necessary to prevent hypoglycemia based on the authors’ experience; however, they did not infer its role in DKA resolution. In an Australian study of 37 patients with SGLT2i diabetic ketosis with or without acidemia and 19 non-SGLT2i DKA in T1D [11], Umapathysivam et al. demonstrated that patients with SGLT2i DKA received a significantly lower insulin dose compared with patients with T1D DKA (44.0 [27.0–82.5] vs. 87.0 [63.0–124.0]; *p* = 0.01), corresponding with a delayed resolution of DKA compared with the T1D group (36 [24–72] vs. 18 [12–27] hours; *p* = 0.002). The concentration of dextrose and the precipitants of DKA were not specified. The authors concluded that the protracted duration of SGLT2i DKA in their cohort may be due to inadequate insulin dosing, and that an increased dextrose infusion rate (or greater dextrose concentration) would allow for increased insulin administration and ketone clearance. In our study consisting similar demographic representation of SGLT2i and non-SGLT2i DKA, 10% dextrose was the dextrose concentration of choice at initiation and was well tolerated without vascular complications, achieving a comparable DKA resolution time. In another retrospective small study comparing six SGLT2i DKA patients with 12 non-SGLT2i DKA patients [10], similar amounts of insulin were used in the SGLT2i DKA group compared to the non-SGLT2i DKA group (94 vs. 93 units, *p* = 0.68) along with a greater amount of 5% dextrose in the SGLT2i DKA group (9.0 vs. 2.8 L, *p* = 0.01), and a longer DKA duration in SGLT2i DKA compared to non-SGLT2i DKA (39 vs. 19 h; *p* < 0.001). Thus, early utilization of 10% dextrose may be warranted for managing SGLT2i DKA to enable higher insulin doses and prompt DKA resolution, with the theoretical benefit of less hypoglycemia.

Another potential mechanism for the comparable resolution times of SGLT2i DKA and non-SGLT2i DKA in our study is the use of VRIII as part of our hospital’s DKA protocol, enabling intensive insulin administration. Whilst there are no head-to-head prospective study comparisons between fixed rate intravenous insulin infusion (FRIII) and VRIII in DKA, VRIII achieved early hyperglycemia correction and DKA resolution without increased hypoglycemia risk in an observational study of 97 patients with DKA [24]. Furthermore, VRIII was associated with a significantly lower incidence of severe hypoglycemia than FRIII (13% vs. 50%; *p* = 0.006) in a retrospective study of 67 patients with DKA (*n* = 53 VRIII and *n* = 14 FRIII) [25]. In our cohort, hypoglycemia occurred in 20% with VRIII, which is lower than the reported 32.5–50% in the literature with FRIII [25,26]. These studies imply that VRIII enables intensive insulin management in DKA with less hypoglycemia than FRIII. Furthermore, VRIII may be particularly beneficial due to the potential rise in glucose levels with 10% dextrose infusions. Hence, treatment of SGLT2i DKA with FRIII may not allow swift escalation of insulin therapy to meet the higher insulin requirement in SGLT2i DKA, and thus potentially prolong DKA duration.

The strengths of our study include detailed characterization of the management, including specification of dextrose and insulin use, which have been seldom reported in the literature limiting conclusions about the optimal management of SGLT2i DKA. Our study demonstrated for the first time that the greater prevalence of intercurrent illnesses is a potential explanation for the clinical cohort differences in higher insulin requirement and longer ICU LOS in SGLT2i DKA compared to non-SGLT2i DKA. Furthermore, in comparison with the existing literature, we demonstrated that the greater insulin requirement in SGLT2i DKA may be met with 10% dextrose and VRIII to achieve a comparable time to DKA resolution without increased adverse events. Additionally, our findings extend the work of Umapathysivam and colleagues [11], emphasizing the importance of intensive management of SGLT2i DKA with greater IV dextrose use with 10% dextrose as the initial dextrose concentration of choice.

Our study has several limitations. In our study, ketosis was defined as BOHB ketones > 0.6 mmol/L corresponding with above the upper limit of normal, which is a lower cut-off than other definitions (e.g., >3.0 mmol/L) [27,28]. However, reports in the literature demonstrate that people taking SGLT2i may become acidemic at a lower degree of ketonemia. In a case series of SGLT2i DKA, multiple cases were found to have acidemia despite relatively mild ketonemia (as low as 0.7 mmol/L) [29]. Hence, we utilized a lower definition of ketonemia to increase the sensitivity of capturing SGLT2i DKA and diabetic ketosis, similar to other studies [7]. However, this may lead to overdiagnosis and dilution of the severity comparisons of DKA. Our study included one SGLT2i DKA and four SGLT2i diabetic ketosis patients managed with subcutaneous insulin without IV insulin. However, the subset analysis of the SGLT2i cohort managed with subcutaneous vs. IV insulin demonstrated the benefits of IV insulin therapy, allowing more intensive insulin therapy to achieve comparable resolution of ketonemia. The disproportionate number of individuals in the subset analysis of four groups (non-SGLT2i DKA without intercurrent illnesses, non-SGLT2i DKA with intercurrent illnesses, SGLT2i DKA without intercurrent illnesses and SGLT2i DKA with intercurrent illnesses) is a limitation of the study. However, our study adds value to the literature, which currently lacks comprehensive evaluation in comparison with intercurrent illnesses, providing unique clinical insights for future study designs. The duration of ICU LOS may be confounded by non-medical confounders, such as lack of bed availability in the general wards leading to increased ICU LOS. However, all SGLT2i and non-SGLT2i group patients were exposed to this environmental confounder in a similar time frame, reducing its heterogeneity. The overall hospital LOS was not evaluated in order to mitigate the effect of non-acute medical factors such as discharge planning, as the rehabilitation waitlist was extended beyond the typical duration during the coronavirus disease pandemic. The study is from a single center, which may limit generalizability. The non-SGLT2i comparator arm was selected based on the time relative to the SGLT2i group rather than by the matching of baseline characteristics or disease severity. However, this temporal proximity matching reflects real-world clinical practice using the same protocols while minimizing variability between care providers.

In conclusion, the greater insulin requirement and ICU LOS in SGLT2i DKA compared to non-SGLT2i DKA may be explained by the greater prevalence of precipitating intercurrent illnesses in SGLT2i DKA and by the greater insulin resistance in T2D, which constituted the majority of cases of SGLT2i DKA. The differences observed between these cohorts reflect underlying demographic distinctions, emphasizing that the patients affected by SGLT2i DKA (predominantly those with T2D) and non-SGLT2i DKA (predominantly those with T1D) represent fundamentally different clinical entities, rather than being solely attributable to the use of SGLT2i medication. High suspicion and investigations for intercurrent illness are warranted in patients presenting with SGLT2i DKA, which typically represent comorbid individuals. Our findings in comparison to the literature imply that in SGLT2i DKA, the classically prolonged requirement for IV insulin infusion may be reduced to a duration similar to non-SGLT2i DKA through intensive management using 10% dextrose and VRIII. This management is likely to have the benefits of reduced total time on IV insulin infusion without increased adverse events in SGLT2i DKA. Guidelines for SGLT2i DKA may need to consider early utilization of 10% dextrose and VRIII. Randomized controlled trials are warranted to evaluate the efficacy of different SGLT2i DKA management strategies.

## Figures and Tables

**Table 1:** Comparison of baseline characteristics and initial biochemistry between SGLT2i and non-SGLT2i groups.

	SGLT2i Group (*n* = 30)	Non-SGLT2i Group (*n* = 60)	*p*-Value
Gender (%)			0.26
Male	19 (63)	29 (48)	
Female	11 (37)	31 (52)	
Age (years) †	53.0 (47.0−62.3)	31.0 (24.3−48.0)	<0.001
BMI (kg/m^2^) †	31.1 (26.5−35.6)	24.2 (21.7−27.2)	<0.001
Duration of diabetes (years) †	6.0 (2.8−16.8)	9.0 (0.0−18.0)	0.80
Types of diabetes (%) ‡			<0.001
Type 1	1 (3)	60 (100)	
Type 2	25 (83)	0 (0)	
LADA	4 (13)	0 (0)	
Premorbid antihyperglycemic agents (%)			0.001
Insulin therapy	12 (40)	45 (75)	
Non-insulin therapy	18 (60)	0 (0)	
None	0 (0)	15 (25)	
HbA1c *	9.8 ± 3.0 % (84 ± 9 mmol/mol)	9.7 ± 2.5 % (83 ± 4 mmol/mol)	0.74
Diabetes complications (%)			
Ischemic heart disease	6 (20)	4 (7)	0.07
Stroke	3 (10)	5 (8)	0.54
Peripheral vascular disease	2 (7)	4 (7)	0.68
Peripheral neuropathy	2 (7)	9 (15)	0.22
Retinopathy	3 (10)	8 (13)	0.47
Chronic kidney disease			
Initial DKA biochemistry			
BGL (mmol/L) †	14.1 (9.9−26.5)	27.1 (23.9−36.6)	<0.001
BOHB Ketones (mmol/L) †	4.1 (3.6−5.5)	6.2 (4.5−7.0)	<0.001
pH †	7.22 (6.98−7.34)	7.10 (6.95−7.21)	0.07
Serum bicarbonate (mmol/L) †	14.0 (11.0−19.0)	9.5 (6.0−13.0)	0.003
Precipitants of DKA (%)			
Infection	18 (60)	22 (37)	0.04
Missed insulin	4 (13)	32 (53)	0.001
Decreased oral intake	8 (27)	6 (10)	0.06
New onset diabetes	0 (0)	13 (22)	0.004
Alcohol	2 (7)	9 (15)	0.32
Acute myocardial infarct	3 (10)	1 (2)	0.11
Steroid therapy	4 (13)	0 (0)	0.01
Pancreatitis	0 (0)	2 (3)	0.55
Surgery	2 (7)	0 (0)	0.11
Ketogenic diet	2 (7)	0 (0)	0.11

*, expressed as mean ± standard deviation; †, expressed as median (25th percentile–75th percentile); ‡, after revision of diabetes type; BGL, blood glucose level; BMI, body mass index; BOHB, Beta-hydroxybutyrate; DKA, diabetic ketoacidosis; HbA1C, hemoglobin A1C; LADA, latent autoimmune diabetes in adults; SGLT2i, sodium–glucose co-transporter 2 inhibitors.

**Table 2:** Comparison of management and outcomes between SGLT2i and non-SGLT2i groups.

	SGLT2i Group (*n* = 30)	Non-SGLT2i Group (*n* = 60)	*p*-Value
Outcomes			
ICU LOS (hours) *	56 (43–101)	40 (23–67)	0.002
Time to pH resolution (hours) *	7 (1–14)	10 (6–13)	0.21
Time to ketonemia resolution (hours) *	22 (15–36)	20 (14–31)	0.91
Recurrent ketonemia (%)	8 (27)	17 (28)	1.00
Mortality (%)	3 (10)	0 (0)	0.04
Insulin administration			
Total insulin dose (units) *	136 (64–217)	89 (55–147)	0.06
Total IV insulin dose (units) *	150 (107–228) ^†^	89 (55–147)	0.004
Total IV insulin duration (hours) *	35 (26–51) ^†^	26 (17–44)	0.08
Total IV insulin over 24 h (units) *	93 (71–137) ^†^	81 (54–111)	0.04
Average IV insulin infusion rate (unit/h) *	3.9 (3.0–5.7) ^†^	3.4 (2.3–4.6)	0.04
IV fluids administration			
5% dextrose (%)	9 (30)	8 (14)	0.09
Amount (liters) ‡	0.4 ± 0.9	0.2 ± 0.6	0.03
Rate (mL/h) *	83 (83–100)	125 (83–146)	0.24
10% dextrose (%)	25 (83)	56 (93)	0.11
Amount (liters) ‡	1.4 ± 1.1	1.9 ± 1.4	0.25
Rate (mL/h) ‡	85 ± 6	82 ± 24	0.57
Total dextrose (g) ‡	157 ± 117	195 ± 135	0.19
NaCl 0.9% (%)	24 (80)	56 (93)	0.08
Amount (liters) *	2.4 (1.2–3.8)	3.0 (2.0–4.5)	0.24
CSL (%)	8 (27)	26 (43)	0.17
Amount (liters) ‡	0.8 ± 1.9	0.9 ± 1.2	0.61
Total amount of IV fluids ‡	5.0 ± 3.2	6.3 ± 2.8	0.06
Complications (%)			
Hypokalemia	20 (67)	37 (62)	0.82
Hypoglycemia	4 (13)	14 (23)	0.52
Vascular complications (e.g., thrombophlebitis)	0 (0)	0 (0)	1.00

*, expressed as median (25th percentile–75th percentile); ^†^, in 25 patients who received IV insulin; ‡, expressed as mean ± standard deviation; BGL, blood glucose level; CSL, compound sodium lactate; DKA, diabetic ketoacidosis; ICU, intensive care unit; IV, intravenous; LOS, length of stay; NaCl, sodium chloride; SGLT2i, sodium–glucose co-transporter 2 inhibitors.

## Data Availability

The data presented in this study are available on request from the corresponding author. The data are not publicly available due to privacy and ethical restrictions.
